# Assessing secular trends in HIV rapid diagnostic test uptake and positivity in Northeast Iran, a country in MENA region; ingredients for target-specific prevention policies

**DOI:** 10.1186/s12879-023-08309-6

**Published:** 2023-05-15

**Authors:** Zahra Yousefli, Najmeh Maharlouei, Maliheh Dadgar Moghaddam, Ali Mohammad Hosseinpour, Roohollah Ghiami

**Affiliations:** 1 Health Policy Research Center, Institute of Health, Shiraz University of Medical Sciences, Shiraz, Iran; 2 Department of Community and Family Medicine, Faculty of Medicine, Mashhad University of Medical Sciences Mashhad, Mashhad, Iran; 3 Department of HIV Care and Prevention, Health Deputy Office, Mashhad University of Medical Sciences, Mashhad, Iran

**Keywords:** Human immunodeficiency Virus, HIV rapid diagnostic test, HIV screening

## Abstract

**Background:**

Iran is amongst the first three countries in Middle East and North Africa (MENA) region where two-thirds of region’s new HIV infections are reported. HIV testing at the population level is key to interrupting the HIV transmission chain. The current study aimed to evaluate the history of HIV rapid diagnostic testing (HIV-RDT) and its correlates in northeast Iran.

**Methods:**

In this cross-sectional study, de-identified records of HIV-RDTs were extracted by the census method from the electronic health information system of 122 testing facilities between 2017 and 2021. Descriptive, bivariate, and multiple logistic regression analyses were performed to identify the factors associated with HIV-RDT uptake and risks and drivers of HIV-RDT positivity, separately among men and women.

**Results:**

Conducting 66,548 HIV-RDTs among clients with a mean age of 30.31 years, 63% female, 75.2% married, and 78.5% with high school education or below, yielded 312 (0.47%) positive results. Test uptake was comparatively low among men and the unmarried sub-population. Prenatal care and high-risk heterosexual intercourse were the most frequent reasons for taking HIV-RDT among women and men, respectively (76% and 61.2%). High-risk heterosexual contact, tattooing, mother-to-child transmission (MTCT), having a partner at risk of HIV infection, and injecting drugs were test seekers’ most reported transmission routes. One-third of the newly-infected female clients were identified through prenatal testing. Multivariate analysis revealed older age at the time of testing (Adjusted Odd Ratio (AOR) = 1.03), divorce (AOR = 2.10), widowhood (AOR = 4.33), education level of secondary school (AOR = 4.67), and unemployment (AOR = 3.20) as significant demographic predictors of positive HIV-RDT (P-value < 0.05). However, clients’ nationality, testing history, duration of HIV exposure, and reported reasons for taking HIV-RDT were not associated with the test result (P-value > 0.05).

**Conclusion:**

Innovative strategies are required to scale up test uptake and positive yields among the key population in the region. The current evidence strongly suggests implementing gender-targeted strategies, according to the differences in demographic and behavioral risk between men and women.

## Background

With a global estimation of 38.4 million people living with Human Immunodeficiency Virus (HIV), 1.5 million new infections, and 650,000 related death in 2021, HIV infection remains a significant public health concern in the world [1].

The Middle East and North Africa (MENA) region comprises Iran and 22 other countries [2]. Despite all the progress made in the region, little is known about HIV and AIDS epidemic in the region being perceived as a black hole [3]. Although the MENA region has been characterized as low HIV prevalence region in the world (less than 0.1%) [2], it has been experiencing the highest increase in the number of newly HIV-infected populations since 2010 [1] with HIV services poorly targeted at key populations [4] and far from UNAIDS 95-95-95 goal to end HIV [2].

Iran with 53,000 HIV infected and 2,200 new cases in 2021 [5], is among the first three countries in the MENA region where two-thirds of the region’s newly HIV-infected cases are reported [6]. Identifying HIV-infected individuals who are unaware of their status has been an area of concern in the HIV care continuum in Iran [7, 8]. It was estimated that only 43% of 53,000 HIV-infected individuals were aware of their HIV-infection status in 2021 in the country [7], conveying the importance of scaling up HIV screening rates at the population level, followed by initiating treatment to interrupt the HIV transmission chain [9].

HIV rapid diagnostic test (HIV-RDT) and counseling, as a screening tool and an essential part of the National Strategic Plan (NSP) in Iran, have been offered to identify and treat newly infected individuals at public health centers (PHC), triangular clinics, and voluntary counseling and testing (VCT) centers [10]. With the help of this strategy, the number of HIV-RDTs has increased about 2.8 times since 2016 in the country [5]. However, as per a national report, the HIV detection rate has not increased accordingly [7], suggesting the need for policymakers to address this gap and to greatly expand and optimize testing coverage. Moreover, a comprehensive report in the MENA region has emphasized the importance of developing gender-specific prevention interventions, based on risk and behavioral differences, to combat the HIV epidemic in the region [11]. Hence, evaluating the history of the HIV-RDT program is critical to recognizing the strengths and weak points for future planning and understanding the HIV risk differences among gender sub-groups. This study aimed to investigate the trend of HIV-RDT uptake and determinant factors of a positive outcome, separately for men and women, in Mashhad and 14 other cities in northeast Iran.

## Methods

### Study design and location

This cross-sectional study was conducted over five years, from March 2017 to March 2022, in Mashhad, a metropolitan city in Iran, and 14 other cities in northeast Iran. These cities contain 115 PHCs and 7 VCTs altogether, supervised by Mashhad University of Medical Sciences, and conduct HIV rapid testing programs based on the national guideline.

### Data source

After obtaining permission and approval from the ethical committee of Mashhad University of Medical Science (Approval code: IR.MUMS.REC.1400.368), de-identified records of all clients who took HIV-RDT were extracted from electronic health information systems (HIS) of Mashhad University of Medical Sciences. All clients whose testing information had been recorded in the electronic HIS were eligible to be included (census method), and those with missing test result information were excluded. The checklist had two parts; demographic information and data on HIV testing. The client’s demographic information including age at testing time, gender, marital status, pregnancy status (if applicable), education level, occupation, and nationality. HIV testing data consisted of self-reported perceived risk behavior (the reason for taking HIV-RDT), the interval between risky behavior and testing, testing history, and the results of the first (screening) and second (confirmatory) HIV-RDT.

### Study population and HIV rapid diagnostic testing

**Fig. 1:**
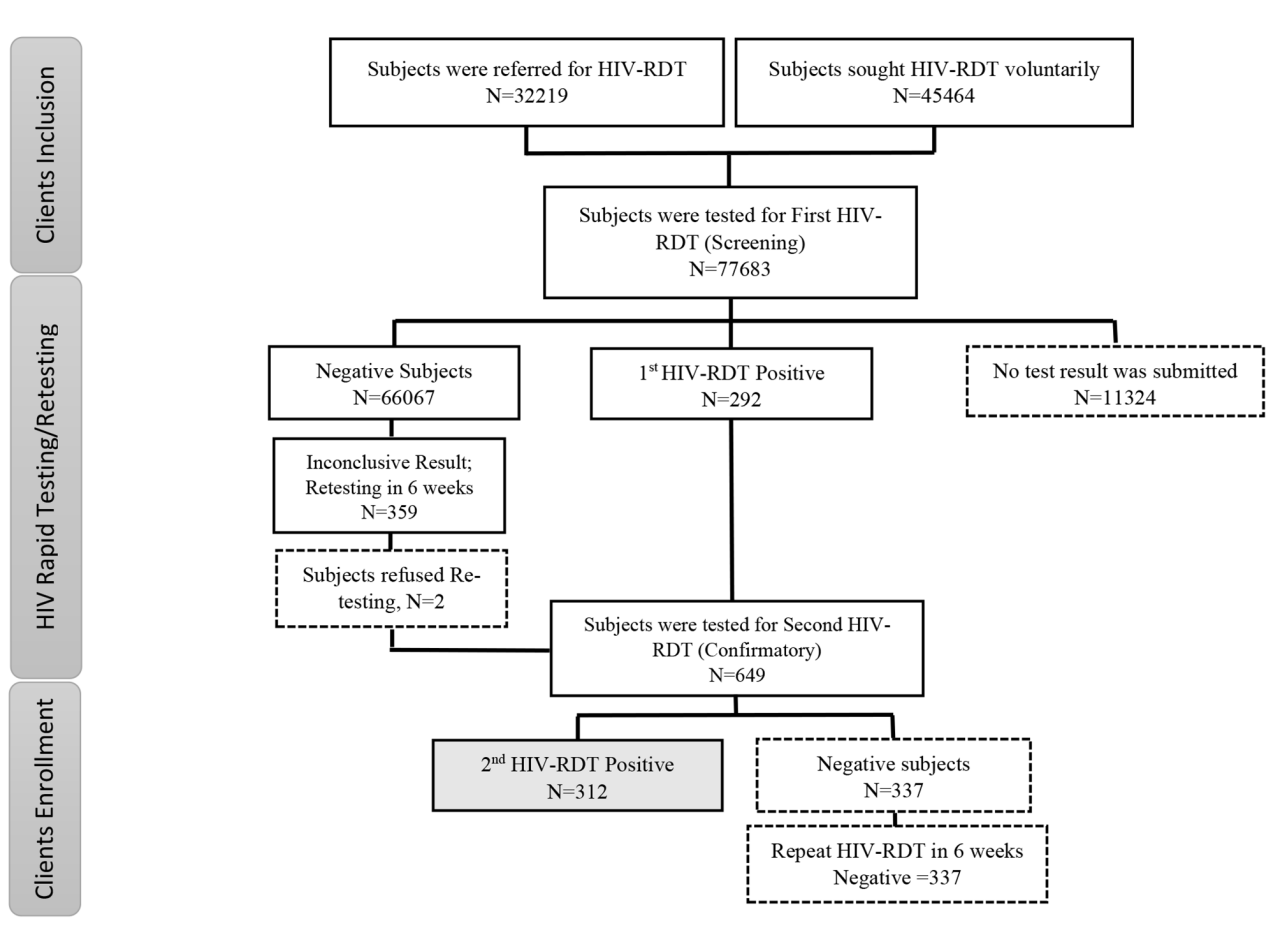
HIV Testing Flowchart

After pre-test counseling and ensuring understanding, informed consent was obtained from the test seekers. All clients, who either sought HIV-RDT voluntarily or had been referred for testing, were asked to complete a codified and de-identified questionnaire with the help of a healthcare provider to collect data on demographic and behavioral characteristics. HIV-RDT is a rapid visual immunoassay for the qualitatively detecting anti-HIV-1 and HIV-2 antibodies in human specimens [12]. In the current study, HIV RDTs were performed through onsite finger-prick whole blood sampling by using an SD HIV-RDT kit (BIOLINE HIV-1/2 3.0) or KHB Diagnostic Kit for HIV (1 + 2) Antibody (Colloidal Gold V2). Following the interpretation of the results, participants were given post-test counseling and a discussion on subsequent retesting or risk reduction plans. Considering the possibility of very early HIV infection (window period), clients with non-reactive or inconclusive screening results were counseled accordingly and retested after six weeks. In addition, a second HIV-RDT was performed as a confirmatory test to ensure an accurate diagnosis for those screened positive. If the confirmatory test resulted negative following a positive screening test (discordant results), the HIV-RDT was repeated within six-week time. All these testing steps were conducted according to World Health Organization guideline [13]. Figure 1 presents a flow diagram of study recruitment and testing outcomes.

### Statistical analysis

Descriptive statistics were utilized to summarize frequencies and percentages for categorical variables and to report mean ± standard deviation (SD) for normally distributed continuous variables. Graphs were created to present HIV-RTD uptake and positivity rate stratified by the study years. The Pearson Chi-square test was used to test differences between categorical variables. Bivariate analysis was conducted to assess the crude associations of demographic and behavioral variables with HIV-RDT positivity and Crude Odds Ratios (OR), with 95% confidence intervals (CI) reported to estimate the strength of the association. Statistical significance was indicated by a P-value less than 0.05.

Independent effects of the demographic and behavioral factors on HIV-RDT positive outcome were reported as Adjusted Odds Ratios (AOR), with 95% CI, produced by Logistic Regression. The regression was performed by adding variables started by inputting the age, gender, marital status, nationality, and education variables, followed by characteristics of HIV test uptake, and then variables related to individual risky behavior. The Hosmer and Lemeshow test assessed the model fitness, and the significant impact on HIV-RDT was based on a Wald P-value of less than 0.05. Statistical analyses were conducted using the Statistical Package for the Social Sciences (SPSS) v. 26 software.

## Results

### Background characteristics and HIV-RDT history of study participants

A total of 66,548 clients received HIV testing in 115 PHC and 7 VCT sites during the study period. Approximately two-thirds of the test takers (62.9%) were female, 75.2% were married, 92.2% were Iranian, and almost half of the clients were homemakers (48.7%). Most test seekers (75%) were below 36 years of age with a mean age of 30.31 ± 9.79, ranging from 3 to 97 years. Levels of education among the majority of the clients (78.3%) were high school diplomas or below.

Pertaining to the interval between risky exposure and testing, only one-third of the clients took HIV-RDT within three months of exposure, and 57.1% of the clients did not recall the exposure time. Table 1 summarizes the study participants’ detailed demographic information and statistics on HIV-RDT uptake.

As shown in Table 4, prenatal care was the most common reason for HIV-RDT uptake in the testing sites (52.1%), followed by high-risk heterosexual intercourse, occupational exposure, and tattooing, accounting for 24%, 6.1%, and 3.4%, in turn. Those clients with other risky behavior including drug injection, men having sex with men (MSM), female sex workers (FSW), and transgenders were the least presented population in the testing facilities. In addition, heterosexual intercourse and prenatal testing contributed 46.7% and 16.7% of positive HIV-RDT among the whole clients, respectively. Table 4 presents reasons for testing and positivity in detail.

**Table 1:** Description of Characteristics of **Total** Study Participants

Variables		HIV RDT- (n = 66,236)	HIV RDT+ (n = 312)	Total (66,548)	P-value	OR^^^ [95% CI]
Age		30.29 ± 9.76	36.23 ± 11.58	30.31 ± 9.79	< 0.001	1.04 (1.036,1.05)^*^
Sex	Female	41,595 (63.0)	144(46.2)	41,739 (62.9)	< 0.001	
	Male	24,429 (37.0)	168(53.8)	24,597(37.1)		1.99(1.59,2.48)^*^
Marital status	Married	49,506 (75.3)	156(51.8)	49,662 (75.2)	< 0.001	
	Never married	13,183 (20.1)	79(26.2)	13,262 (20.1)		1.90(1.45,2.49)^*^
	Divorced	2455 (3.7)	39(13.0)	2494 (3.8)		5.04(3.54,7.18)^*^
	Widowed	601 (0.9)	27(9.0)	628 (1.0)		14.26(9.40,21.62)^*^
Occupation	Homemaker	20,845 (48.9)	58 (25.0)	20,903 (48.7)	< 0.001	
	Unemployed	2906 (6.8)	78 (33.6)	2984 (7.0)		9.65(6.85,13.58)^*^
	Employee	16,216 (38.0)	79 (34.1)	16,295 (38.0)		1.75(1.25,2.46)^*^
	Self-employed	2693 (6.3)	17 (7.3)	2710 (6.3)		2.27(1.32,3.90)^*^
Education level	Illiterate	3019 (4.6)	26 (8.3)	3045 (4.6)	0.001	
	Primary school	11,130 (16.9)	59 (18.9)	11,189 (16.9)		0.62(0.39,0.98)^*^
	Secondary school	15,081 (22.8)	82 (26.3)	15,163 (22.9)		0.63(0.41,0.98)^*^
	High school and diploma	22,450 (34.0)	98 (31.4)	22,548 (34.0)		0.51(0.33,0.78)^*^
	University degree	14,335 (21.7)	47 (15.1)	14,382 (21.7)		0.38(0.24,0.62)^*^
Nationality	Iranian	60,898 (92.2)	278 (89.1)	61,176 (92.2)	0.042	
	Non-Iranian	5148 (7.8)	34 (10.9)	5182 (7.8)		1.45(1.01,2.07)^*^
Testing History	First Time	62,928 (95.3)	297 (95.2)	63,225 (95.3)	0.940	
	Repeated	3115 (4.7)	15 (4.8)	3130 (4.7)		1.02(0.61,1.72)
Referral Type	Referred Client	20,901 (31.6)	129 (41.3)	21,030 (31.7)	< 0.001	
	Volunteer	45,145 (68.4)	183 (58.7)	45,328 (68.3)		0.66(0.52,0.82)^*^
Duration from First Exposure	< 3 months	10,089 (30.5)	29 (20.4)	10,118 (30.5)	0.009	
	≥ 3 months	22,971 (69.5)	113 (79.6)	23,084 (69.5)		1.71(1.14,2.58)^*^

Data were reported as mean ± standard deviation and n (%).

Mann–Whitney U and Chi-Square tests were used.

^Crude Odds Ratio resulted from Bivariate analysis.

*Considered statistically significant.

### Background characteristics and HIV-RDT history of male clients

As illustrated in Table 2, the mean age among men was 32.46 ± 11.02 years. 50% of the male test takers were married, 16.2% were unemployed, and one-third had university degrees. Male clients were more likely to seek tests voluntarily (90.7%), and after a 3-month exposure time (85%).

High-risk heterosexual intercourse was reported as a testing reason by 61.1% of the male clients in the testing sites, followed by occupational exposure (9.0%), tattooing (6.9%), and outside facility HIV testing campaigns (4.9%).

Following heterosexual intercourse yielded 60.2% of the positive results, and tattooing, MTCT, TB, and drug injection yielded 8.8%, 6.2%, 5.3%, and 3.5% of positive HIV-RDT in the male population, respectively.

**Table 2:** Description of Characteristics of **Male** Study Participants

Male		HIV RDT- (n = 24,429)	HIV RDT+ (n = 168)	Total 24,597	P-value	OR^^^ [95% CI]
Age		32.42 ± 11.00	37.86 ± 11.66	32.46 ± 11.02	< 0.001	1.04 (1.03,1.05)^*^
Marital status	Married	12,161 (50.1)	69 (42.6)	12,230 (50.0)	< 0.001	
	Never married	10,644 (43.8)	63 (38.9)	10,707 (43.8)		1.04(0.74,1.47)
	Divorced	1235 (5.1)	19 (11.7)	1254 (5.1)		2.71(1.63,4.52)^*^
	Widowed	237 (1.0)	11 (6.8)	248 (1.0)		8.18(4.27,15.66)^*^
Occupation	Homemaker	61 (0.5)	0 (0)	61 (0.5)	< 0.001	
	Unemployed	2101 (15.9)	56 (45.2)	2157 (16.2)		4.27 (2.17,8.4)^*^
	Employee	9459 (71.5)	58 (46.8)	9517 (71.3)		0.00
	Self-employed	1603 (12.1)	10 (8.1)	1613 (12.1)		0.98 (0.5,1.93)
Education level	Illiterate	870 (3.6)	11 (6.5)	881 (3.6)	< 0.001	
	primary school	3296 (13.5)	35 (20.8)	3331 (13.5)		0.84(0.43,1.66)
	Secondary school	4775 (19.5)	51 (30.4)	4826 (19.6)		0.85(0.44,1.63)
	High school and diploma	8038 (32.9)	43 (25.6)	8081 (32.9)		0.42(0.22,0.82)^*^
	University Degree	7447 (30.5)	28 (16.7)	7475 (30.4)		0.30(0.15,0.60)^*^
Nationality	Iranian	23,459 (96.0)	151 (89.9)	23,610 (96.0)	< 0.001	
	Non-Iranian	970 (4.0)	17 (10.1)	987 (4.0)		2.72(1.64,4.51)^*^
Testing History	First Time	23,400 (95.8)	160 (95.2)	23,560 (95.8)	0.724	
	Repeated	1029 (4.2)	8 (4.8)	1037 (4.2)		1.14(0.56,2.32)
Referral Type	Referred Client	2238 (9.2)	60 (35.7)	2298 (9.3)	< 0.001	
	Volunteer	22,191 (90.8)	108 (64.3)	22,299 (90.7)		0.18(0.13,0.25)^*^
Duration from First Exposure	< 3 months	1933 (15.0)	13 (17.6)	1946 (15.0)	0.542	
	≥ 3 months	10,933 (85.0)	61 (82.4)	10,994 (85.0)		0.83(0.46,1.51)

Data were reported as mean ± standard deviation and n (%).

Mann–Whitney U and Chi-Square tests were used.

^Crude Odds Ratio resulted from Bivariate analysis.

*Considered statistically significant.

### Background characteristics and HIV-RDT history of female clients

Table 3 shows that the mean age of female test seekers was 29.06 ± 8.74 years. 90% were married, 70.6% were pregnant, 90% were Iranian, 70.6% were homemakers, and 16.5% had university degrees. Women were referred for HIV-RDT more than men, apart from prenatal visits. (15.7% vs. 9.3%, p-value < 0.001).

As illustrated in Table 4, prenatal care (77.5%), high-risk heterosexual intercourse (5.8%), occupational exposure (4.7%), and having a partner who is at HIV risk (3.5%) were the first four common reasons for taking HIV-RDT among females. Moreover, prenatal care accounted for 33.0% of the positive yields, followed by heterosexual intercourse at 29.6%, having a partner at HIV risk at 7.8%, TB at 5.2%, and physician orders at 5.2% in the female population (Table 4).

**Table 3:** Description of Characteristics of **Female** Study Participants

Female		HIV RDT- (n = 41,595)	HIV RDT+ (n = 144)	Total 41,739	P-value	OR^^^ [95% CI]
Age		29.4 ± 8.72	34.33 ± 11.23	29.06 ± 8.74	< 0.001	1.05 (1.03,1.06)^*^
Marital status	Married	37,332 (90.1)	87 (62.6)	37,419 (90.0)	< 0.001	
	Never married	2533 (6.1)	16 (11.5)	2549 (6.1)		2.71(1.59,4.63)^*^
	Divorced	1220 (2.9)	20 (11.5)	1240 (3.0)		7.03(4.31,11.48)^*^
	Widowed	364 (0.9)	16 (11.5)	380 (0.9)		18.86(10.9,32.5)^*^
Pregnancy Status	Yes	29,421 (70.7)	39 (27.1)	29,460 (70.6)	< 0.001	
	No	12,174(29.3)	105 (72.9)	12,279 (29.4)		6.51(4.50,9.40)^*^
Occupation	Homemaker	20,781 (70.6)	58 (53.7)	20,839 (70.6)	< 0.001	
	Unemployed	803 (2.7)	22 (20.4)	825 (2.8)		9.82(5.98,16.1)^*^
	Employee	6754 (23.0)	21 (19.4)	6775 (22.9)		1.11(0.68,1.84)
	Self-employed	1090 (3.7)	7 (6.5)	1097 (3.7)		2.30(1.05,5.05)^*^
Education level	Illiterate	2149 (5.2)	15 (10.4)	2164 (5.2)	0.039	
	Primary school	7831 (18.8)	24 (16.7)	7855 (18.8)		0.44(0.23,0.84)^*^
	Secondary school	10,302 (24.8)	31 (21.5)	10,333 (24.8)		0.43(0.23,0.80)^*^
	High school and diploma	14,405 (34.7)	55 (38.2)	14,460 (34.7)		0.55(0.31,0.97)^*^
	University degree	6883 (16.6)	19 (13.2)	6902 (16.5)		0.40(0.20,0.78)^*^
Nationality	Iranian	37,421 (90.0)	127 (88.2)	37,548 (90.0)	0.48	
	Non-Iranian	4174 (10.0)	17 (11.8)	4191 (10.0)		1.20(0.72,1.99)
Testing History	First Time	39,511 (95.0)	137 (95.1)	39,648 (95.0)	0.935	
	Repeated	2084 (5.0)	7 (4.9)	2091 (5.0)		0.97(0.45,2.07)
Referral Type	Referred Client	18,658 (44.9)	69 (47.9)	18,727 (44.9)	0.461	
	Volunteer	22,937 (55.1)	75 (52.1)	23,012 (55.1)		0.88(0.64,1.23)
Duration from First Exposure	< 3 months	8154 (40.4)	16 (23.5)	8170 (40.4)	0.005	
	≥ 3 months	12,024 (59.6)	52 (76.5)	12,076 (59.6)		2.20(1.26,3.86)^*^

Data were reported as mean ± standard deviation and n (%).

Mann–Whitney U and Chi-Square tests were used.

^Crude Odds Ratio resulted from Bivariate Analysis.

*Considered statistically significant.

**Table 4:** Reasons for Taking HIV-RDT of Study Participants

	Total			Male			Female		
	**HIVRDT-**	**HIVRDT+**	**Total**	**HIVRDT-**	**HIVRDT+**	**Total**	**HIV RDT-**	**HIVRDT+**	**Total**
Prenatal care	29794 (52.2)	38 (16.7)	29832 (52.1)	-	-	-	29776(77.7)	38(33.0)	29814(77.5)
High-risk heterosexual intercourse	13643 (23.9)	102(44.7)	13745(24.0)	11447(61.1)	68(60.2)	11515(61.1)	2191(5.7)	34(29.6)	2225(5.8)
Physician’s order due to sign and symptom	1076 (1.9)	8 (3.5)	1084(1.9)	612(3.3)	2(1.8)	614(3.3)	464(1.2)	6(5.2)	470(1.2)
Occupational exposure	3498 (6.1)	3(1.3)	3501(6.1)	1702(9.1)	0(0)	1702(9.0)	1793(4.7)	3(2.6)	1796(4.7)
Partner of a person at HIV risk^¶^	1451(2.5)	10(4.4)	1461(2.5)	132(0.7)	1(0.9)	133(0.7)	1319(3.4)	9(7.8)	1328(3.5)
Tuberculosis Co-infection	816 (1.4)	12 (5.3)	828 (1.4)	374(2.0)	6(5.3)	380(2.0)	442(1.2)	6(5.2)	448(1.2)
Tattooing	1921 (3.4)	11 (4.8)	1932 (3.4)	1290(6.9)	10(8.8)	1300(6.9)	629(1.6)	1(0.9)	630(1.6)
Blood transfusion	232 (0.4)	1 (0.4)	233 (0.4)	77(0.4)	0(0)	77(0.4)	155(0.4)	1(0.9)	156(0.4)
Drug injection within one year	17 (0.0)	5 (2.2)	22 (0.0)	17(0.1)	4(3.5)	21(0.1)	0(0)	1(0.9)	1(0.0)
MSM	5 (0.0)	2 (0.9)	7 (0.0)	5(0.0)	2(1.8)	7(0.0)	--	-	-
Partner of an HIV-infected one	17 (0.0)	1 (0.4)	18 (0.0)	6(0.0)	0(0)	6(0.0)	11(0.0)	1(0.9)	12(0.0)
Having STD	119 (0.2)	0 (0)	119 (0.2)	13(0.1)	0(0.0)	13(0.1)	106(0.3)	0(0)	106(0.3)
MTCT	53 (0.1)	12 (5.3)	65 (0.1)	25(0.1)	7(6.2)	32(0.2)	28(0.1)	5(4.3)	33(0.1)
FSW	4 (0.0)	0 (0)	4 (0.0)	-	-	-	4(0.0)	0(0)	4(0.0)
Cupping	140 (0.2)	0 (0)	140 (0.2)	86(0.5)	0(0)	86(0.5)	54(0.1)	0(0)	54(0.1)
History of Prison or Addiction Rehab Center	271 (0.5)	4 (1.8)	275 (0.5)	255(1.4)	4(3.5)	259(1.4)	16(0.0)	0(0)	16(0.0)
Substance abuse	320 (0.6)	5 (2.2)	325 (0.6)	296(1.6)	3(2.7)	299(1.6)	24(0.1)	2(1.7)	26(0.1)
Unsanitary dental procedure	175 (0.3)	1 (0.4)	176 (0.3)	64(0.3)	1(0.9)	65(0.3)	111(0.3)	0(0.0)	111(0.3)
Person's request	825 (1.4)	0 (0)	825 (1.4)	515(2.7)	0(0)	515(2.7)	310(0.8)	0(0)	310(0.8)
Unsanitary medical procedures	28 (0.0)	1 (0.4)	29 (0.1)	15(0.1)	0(0)	15(0.1)	13(0.0)	1(0.9)	14(0.0)
Having an HIV-infected family member	35 (0.1)	0 (0)	35 (0.1)	17(0.1)	0(0)	17(0.1)	18(0.0)	0(0)	18(0.0)
Community-based HIV Testing Campaigns	1397 (2.4)	1 (0.4)	1398 (2.4)	925(4.9)	1(0.9)	926(4.9)	471(1.2)	0(0)	471(1.2)
Hepatitis Co-infection	31 (0.1)	0 (0)	31 (0.1)	8(0.0)	0(0.0)	8(0.0)	23(0.1)	0(0)	23(0.1)
Dormitory Residency	212 (0.4)	1 (0.4)	213 (0.4)	134(0.7)	1(0.9)	135(0.7)	78(0.2)	0(0)	78(0.2)
Needle or sharp-pointed things sticking	409 (0.7)	1 (0.4)	410 (0.7)	259(1.4)	0(0)	259(1.4)	150(0.4)	1(0.9)	151(0.4)
Child Labor	99 (0.2)	0 (0)	99 (0.2)	68(0.4)	0(0)	68(0.4)	31(0.1)	0(0)	31(0.1)
Any suspicious contact	112 (0.2)	1 (0.4)	113 (0.2)	53(0.3)	1(0.9)	54(0.3)	59(0.2)	0(0)	59(0.2)
Rape	25 (0.0)	0 (0)	25 (0.0)	7(0.0)	0(0)	7(0.0)	18(0.0)	0(0)	18(0.0)
Transgender	2 (0.0)	0(0)	2 (0.0)	2(0.0)	0(0)	2(0.0)	0(0.0)	0(0)	0(0.0)
Two or more risks of the above	354 (0.6)	8 (3.5)	362 (0.6)	314(1.7)	2(1.8)	316(1.7)	40(0.1)	6(5.2)	46(0.1)
P value	< 0.001			< 0.001			< 0.001		
AOR [95% CI]	1(1,1)			1(1,1)			1(1,1)		

AOR: Adjusted Odds Ratio, MTCT: mother-to-child transmission, MSM: Men have Sex with Men, FSW: Female Sex Workers, STD: Sexuality Transmitted Diseases

¶ Partner characteristics included illicit drug use, history of incarceration, the concurrence of sexual relationships, and occupational risks.

## HIV-RDT frequency and positivity

**Fig. 2:**
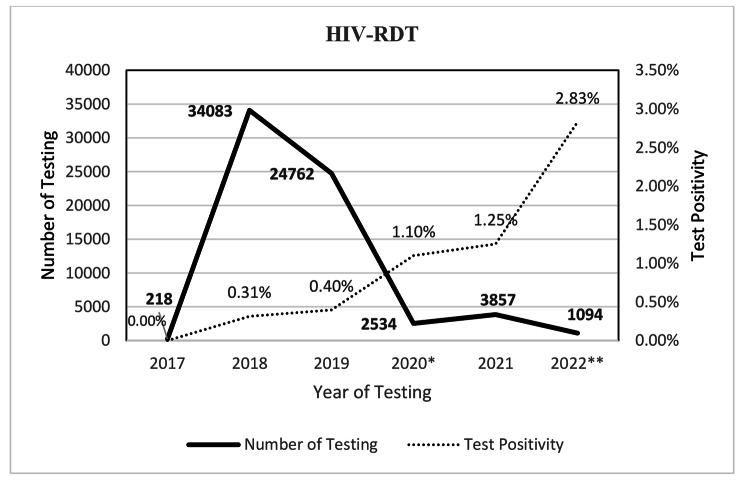
HIV-RDT Frequencies per Year * Start of COVID-19 Pandemic **The first 3 months of 2022

Figure 2 depicts the frequency of HIV testing and test positivity each year during the study period. Over five years, conducting 66,548 HIV-RDTs yielded 312 (0.47%) positive results, 168 (53.8%) of which were men. The number of HIV testing experienced a sharp reduction by 90% in 2020 (P-value < 0.001). Despite an overall downward trend in HIV testing, the test positivity rate increased during that time (P-value < 0.001).

**Fig. 3:**
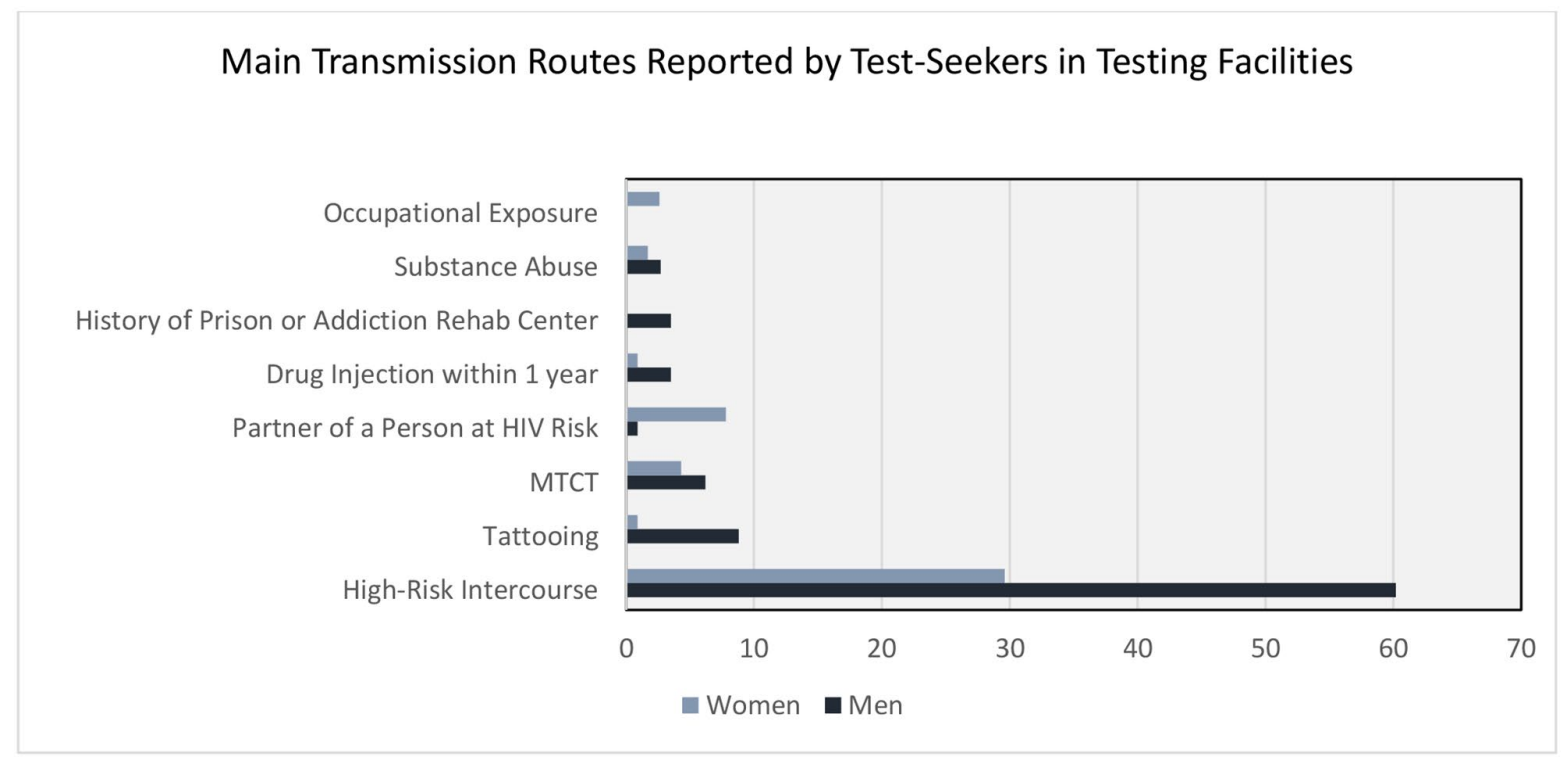
Main Transmission Routes Reported by Test-Seekers in Testing Facilities in percent (%) MTCT: mother-to-child transmission

Although many newly infected individuals were identified through prenatal care, physicians’ orders, or provider-initiated testing in which the transmission routes were not characterized, the main transmission routes reported by the clients were depicted in Fig. 3.

## Bivariate analyses

The results of the bivariate analysis have been illustrated in Table 1 for the whole population. The odds ratio of positive HIV-RDT among men was approximately two times greater than that among women (OR = 1.99[1.59,2.48], P-value < 0.001). Moreover, older age at the testing time (OR = 1.04[1.036,1.05]), never married status (OR = 1.90[1.45,2.49]), divorce (OR = 5.04[3.54,7.18]), widowhood (OR = 14.26[9.40,21.62]), non-Iranian nationality (OR = 1.45[1.01,2.07]) were the other factors associated with positive HIV-RDT (P-value < 0.05). However, the volunteer test seekers (OR = 0.66[0.52,0.82]), and those with any level of education relative to illiteracy, and were less likely to be diagnosed as positive (OR < 1, P-value < 0.05).

Those test takers reported more than 3-month time from HIV exposure accounted for approximately 80% of positive results. (OR = 1.71(1.14,2.58), P-value = 0.009). In terms of occupation, although unemployed subjects comprised only 7% of the test takers, they accounted for 33.6% of the positive results (OR = 9.65(6.85,13.5), P-value < 0.001).

## Multivariate analysis

### Factors associated with positive HIV-RDT outcome

As sown in Table 5, logistic regression analysis revealed that 1-year older age at the time of testing could slightly increase the odds of HIV-RDT positivity by 1.03 times ([95%CI: 1.01,1.05], P-value = 0.001). The most remarkable predictor in terms of marital status was widowhood, which significantly increased test positivity by approximately four times ([95%CI:1.6,11.94], P-value < 0.001). Another predictor was the level of education, with the highest risk at the secondary school level (AOR: 4.67[95%CI:1.44,15.11], P-value = 0.01). Unemployment status could increase the odds of HIV-RDT positive results by about three times ([95%CI:1.59,6.45], P-value 0.001). However, the clients’ gender, nationality, testing history, duration of exposure, and reason for testing could not predict the positive result of the tests in the final multivariate analysis (P-value > 0.05).

### Factors associated with positive HIV-RDT among men

The multivariate analysis showed that 1-year older age at the time of testing could increase HIV-RDT positivity by 1.05[1.02,1.08] times among men (P-value < 0.001). Moreover, male clients were significantly less likely to be detected as positive when seeking tests voluntarily (AOR = 0.38[0.17,0.82], P-value = 0.014).

### Factors associated with positive HIV-RDT among women

Multiple regression revealed that widowed and divorced female clients had approximately three- and seven-time higher likelihood of a positive result of HIV-RDT, respectively (AOR = 2.71[95%CI:1.07,6.87] and 7.07[95%CI:1.96,25.54], P-value < 0.005). Regarding occupation, unemployment was accompanied by a 2.69[95%CI:1.14,6.34] times higher HIV-RDT positivity rate (P-value = 0.024). Moreover, non-pregnant women were more likely to be diagnosed as positive compared to pregnant clients (AOR = 5.04[95%CI:1.20,21.13], P-value = 0.027). However, unlike men, age was not significantly associated with test positivity. (P-value > 0.05).

**Table 5:** Logistic Regression of Clients’ Independent Characteristics and Positive HIV-RDT Outcome

Variable		TOTAL		MALE		FEMALE	
		AOR [95%CI]	Wald P-value	AOR [95%CI]	Wald P-value	AOR [95%CI]	Wald P-value
Age		1.03(1.01,1.05)	0.006	1.05(1.02,1.08)	0.001	1(0.97,1.04)	0.847
Sex	Female						
	Male	1.50(0.86,2.61)	0.152				
Marital status	Married						
	Never married	1.81(0.97,3.35)	0.060	2.06(0.92,4.62)	0.081	2.09(0.78,5.57)	0.140
	Divorced	2.10(1.04,4.25)	0.040	2.04(0.66,6.27)	0.214	2.71(1.07,6.87)	0.036
	Widowed	4.33(1.57,11.94)	0.005	4.64(0.88,24.50)	0.071	7.07(1.96,25.54)	0.003
Pregnancy Status	Yes						
	No	7.76(2.51,23.97)	0.000			5.04(1.20,21.13)	0.027
Occupation	Homemaker			0	0.999		
	Unemployed	3.20(1.59,6.45)	0.001	2.01(0.75,5.44)	0.168	2.69(1.14,6.34)	0.024
	Employee	3.20(1.59,6.45)	0.520	0.54(0.21,1.40)	0.205	0.78(0.38,1.62)	0.507
	Self-employed	0.81(0.42,1.55)	0.960			0.44(0.10,2.06)	0.300
Education level	Illiterate		0.015				
	Primary school	1.85(0.57,6.0)	0.304	1.93(0.34,10.9)	0.454	1.35(0.28,6.59)	0.712
	Secondary school	4.67(1.44,15.11)	0.010	4.94(0.9,27)	0.065	3.06(0.63,14.96)	0.166
	High school and diploma	3.03(0.92,10.03)	0.069	2.05(0.35,12)	0.427	2.72(0.56,13.1)	0.213
	University degree	2.10(0.60,7.38)	0.248	1.51(0.24,9.67)	0.665	1.96(0.37,10.34)	0.425
Testing History	First Time						
	Repeated	0.66(0.25,1.70)	0.389	1.05(0.31,3.55)	0.932	0.51(0.11,0.2.29)	0.378
Nationality	Iranian						
	Non-Iranian	1.60(0.73,3.46)	0.238	1.61(0.46,5.64)	0.460	1.58(0.58,4.28)	0.369
Referral Type	Referred Client						
	Volunteer	0.53(0.32,0.89)	0.017	0.38(0.17,0.82)	0.014	0.67(0.33,1.37)	0.273
Duration from First Exposure	< 3 months						
	≥ 3 months	1.20(0.70,2.08)	0.389	1.46(0.59,3.60)	0.416	0.95(0.47,1.94)	0.887
Reasons for Taking HIV-RDT		1(1,1)	0.695	1(1,1)	0.112	1(1,1)	0.497

Multicollinearity did not exist in the regression model. Homogeneity assumption of the variance was met.

AOR: Adjusted Odds Ratio, CI: Confidence Interval.

P-value < 0.05 is considered statistically significant.

## Discussion

We evaluated the factors associated with HIV-RDT uptake and determinants of positive results among male and female test takers, aged 3–97 years, seeking HIV-RDT to check their status or being referred for testing, in 122 testing sites located in 15 cities in northeast Iran.

We found a sharp decrease in the number of HIV tests performed in 2020 compared to 2019. Similar findings with an overall reduction of 50% in HIV testing were reported in the WHO Eastern Mediterranean region, where Iran is located [14], 26.19% reduction in Europe, 34.67% in Africa, 39.41% in Asia, and 44.62% in Latin America [15]. The explanation could be the start of the COVID-19 pandemic and the stay-at-home period, which aggravated testing obstacles due to fear of contracting COVID-19, as a life-threatening disease, COVID-19-related stigma, reallocating budget and human resources to the COVID-19 care provision [16–18]. Moreover, this study’s significant upward trend in positive test results during the COVID-19 pandemic may suggest either increasing HIV prevalence or targeted screening strategies prioritizing the key population, the latter of which has also been reported in a review of 44 countries in 4 continents during COVID-19 pandemic, ranging from 2.2% increases in positive yields in African countries to 44% in European countries [15].

According to the current findings, HIV-RDTs uptake among women was almost twice as high as among men. This seems to be a consistent finding across various studies [19–22]. It could be partially attributable to the several health services devoted to women providing them with the opportunities to take HIV-RDT. The same finding has been reported in Senegal, taking reproductive health care to explain much of the difference in HIV test uptake between the two genders [23]. Men are more engaged in work and may have less access to testing facilities, blaming inconvenient clinic hours that seeking health services means work absence, lost wages, and poverty [24]. Other reported barriers to male test seeking were stigma, confidentiality concerns, distance to the facility, and perceived such services as weakness or feminine compromising their masculinity [19, 25], resulting in a 1.4 times higher likelihood of a late diagnosis of HIV infection in men compared to women [26]. Indeed, the four times higher participation of men in testing conducted through community-based HIV testing campaigns in the current study may emphasize the importance of HIV testing outside conventional facilities to reach higher testing coverage among men. This is in agreement with findings previously reported from outreach testing programs and event-based testing in Tanzania [27] or self-testing at home in Malawi [28], as examples of community-based testing, which is strongly recommended by WHO to be implemented to scale up testing coverage [9].

Low male testing uptake can increase HIV transmission to their female partners, as well [25]. According to a study on married women in Iran, 20% of the risks of HIV infection were imposed by their spouses on them [29]. This could explain why having a sexual partner at risk of contracting HIV was among the main reasons for HIV-RDT uptake and positivity among women in the current study.

Despite taking fewer HIV-RDTs, men were approximately two times more likely to be identified as positive than women in bivariate analysis. Since most women in our study took HIV-RDT as prenatal care (70.6%), with probably low underlying risks, there were less likely than men to be identified as positive. Female clients might have also received more HIV-related education in prenatal visits, resulting in less inclination to have hazardous behaviors [24]. The association between HIV infection and the male gender has been previously reported as 2.18 times greater odds in a nationwide systematic review in Iran [24]. Given the higher risk, less testing, and late diagnosis among men, policymakers should provide male-centered approaches to overcome barriers to male testing engagement, such as flexible clinic hours, mobile testing sites, and promoting home testing and self-testing [25].

In the present study, older age was positively associated with higher HIV-RDT positivity, particularly among men. Young adults and adolescents are often less presented for HIV testing or using related services in Iran and MENA region [11, 26] or African countries [22, 30] decreasing the chance of being identified as positive compared to older men or women. This could be because older men and women might enjoy more social and economic power with higher confidence in seeking tests [23].

Our findings showed non-married population’s engagement in HIV-RDT was relatively low. Married women in other studies had higher odds of being ever tested for HIV, attributed to testing for pre-marriage or prenatal care [31, 32]. More importantly, multivariate analysis showed that divorce and widowhood were factors related to a higher probability of positive HIV-RDT, particularly among women. As previously published, formerly married women had a significantly higher prevalence of HIV compared to currently-married or never-married women [33, 34]. The disparity in HIV status by women’s matrimony was consistent with a joint report by WHO and UNAIDS, which was attributed to differences in social treatment or treatment with disdain of these groups of women [35]. They may engage in hazardous sexual activities with a high rate of partner change or might be sexually exploited through temporary marriage or offering financial support that imposes more infection risk on them [34]. Despite higher test uptake and relatively lower HIV infection among married women in our study, they are considered a vulnerable group in terms of HIV infection [11]. It is not unlikely that widowed or divorced women became infected during the marriage or even before that [34]. Given that drug injection is still the main route of HIV transmission in Iran [36], and nearly half of the injecting drugs users living in Iran (with male predominance) are married and a third are engaged in extramarital sexual relationships, drug users pass the virus to their female partners by sexual intercourses [37]. Therefore, married women constitute the bridge population in the HIV transmission chain in the MENA region and Iran [11], and interrupting the interaction between drug injection and sexual contact by targeting married women is key to halting the epidemic progression [11]. In accordance with the existing literature mentioned a changing pattern in transmission route from intravenous drug injection to sexual contact in Iran [36], the majority of our positive clients had reported heterosexual contact as their transmission route and one-third of the female clients with positive test result were pregnant women identified thorough prenatal testing. To address these issues, multisectoral prevention interventions at national levels are required which should not be gender-neutral as previously mentioned by Dworkin et al. [20].

Levels of education were of great significance in tests seeking behavior and positive test results. In line with our findings, the odds of being ever tested for HIV rose along with an increase in the levels of education from illiteracy to school education in some African countries [32, 38, 39]. More importantly, lower education levels compared to university education were associated with a higher positivity rate in the current study. This conveys the importance of education in improving HIV-related knowledge, utilization of health services, and reducing HIV transmission [38, 39]. Given the evidence, we suggest providing quality education to improve life skills, particularly secondary and high school-based programs, with health and sex education related to HIV risk factors, transmission routes, and prevention [11].

Our finding supports less test-seeking behavior and a higher likelihood of HIV transmission among unemployed clients. Consistent with this, the state of being employed has been suggested as a significant factor in reducing HIV transmission and better HIV prevention outcomes [40, 41]. In a study conducted in France, unemployment status was reported to be associated with late testing among MSM [42], and in Italy, it was associated with never being tested among women [43]. Consistent evidence shows that men in professional industries reported 66% less unprotected heterosexual intercourse and alcohol consumption [44], and irregular or unstable employment is associated with increased partners and sexual events [45].

Despite the measures taken to reduce vertical transmission of HIV in Iran, leading to a decrease in the absolute number of vertically infected, MTCT is still a concern in the HIV care continuum [2]. Previous studies attributed the statistics to late diagnosis of the disease, after natural childbirth, late prophylaxes for newborns, and a lack of awareness and education for pregnant women in Iran [46]. Additionally, increasing numbers of sexually transmitted HIV infection among women in the past decade has led to an increased number of women living with HIV and consequently infected newborns [47]. Although Iran is amongst the countries with relatively high testing rates for pregnant women, as nearly half number of our testing was devoted to prenatal care, the coverage levels were not reported any higher than 65% in previous studies [48].

First-time test takers did not show a significantly higher chance of being identified as positive compared to re-testers in the current study. In contrast, Martelli et al., in Tanzania, Africa reported significantly higher positive yield in first-time testers, which was attributed to a lack of risk perception in first-time testers [27]. However, according to our finding both first-time testers and re-tester might benefit from HIV-RDT and should be targeted in the region.

In our study, the population with hazardous behavior was less likely to seek HIV-RDT in PHC/VCTs. According to previous studies in Iran and other countries, those at the highest risk such as FSW and MSM are reluctant to be tested for HIV or even report their testing results in surveys due to some cultural constraints, fear of criminalization, and social rejection [23, 49]. Another explanation is that, in Iran, the populations at higher risk such as prisoners, people who inject drugs, and dormitory residents, might be approached directly and actively tested for HIV through active case finding (ACF) programs in prisons, addiction recovery centers, dormitories, and other enclosed facilities [50, 51], the results of which may not be submitted at the same dataset utilized in this study. Given the barriers, HIV self-testing kits have been introduced in some countries in the MENA region to encourage and enable more people to test at home by lifting some of the aforementioned testing barriers [3]. Although these kits have been available in Iran since 2018, they should be more acknowledged and integrated into the prevention program, particularly for critical populations [3].

### Limitations

One limitation of our study is missing information in HIS due to the result being incompletely submitted in HIS or presented in a different dataset in the case of ACF. Another limitation was the study duration, which included the COVID-19 pandemic, which might affect the number of clients. Moreover, this study was a cross-sectional study with a sampling procedure based on the Census (gathering information about every member of the population). This caused an imbalance in population size between the compared groups, making us unable to fully measure confounding factors. Thus, the results are not supposed to show the causation or predict the main outcome (positive HIV-RDT) based on demographic or behavioral variables.

### Strength points

This study is unique as no other study has analyzed the database of the HIS affiliated with other medical universities in Iran. Furthermore, we analyzed data from five years to be able to depict a reliable trend. Additionally, we used data from all centers including urban and rural areas with a large population size, hence we can claim that the method and results of this study can be extrapolated to Iran to expand the coverage.

## Conclusion

This study provides evidence on HIV-RDT uptake, drivers of HIV transmission, and demographic and behavioral risk determinates of positive HIV-RDT outcomes. These findings highlighted the need for target-specific interventions in the area which can have implications for policymakers to expand the testing coverage. Despite a downward trend in the number of testing over five years, we found an upward trend in positive yield. The study showed a relatively low HIV-RDT uptake among men and unmarried populations and promoting community-based testing outside conventional facilities with flexible hours, such as home, mobile or self-testing can lift the testing barriers among men. Moreover, higher HIV-RDT positivity rates among men, divorced, widowed, unemployed, and those with a high-school education or below ask for specific innovative preventive strategies to focus on these populations. Since married women consider a bridge population in HIV transmission, addressing HIV-related knowledge insufficiency, empowering them through life skill school-based education, and scaling up prenatal testing might help better combat the epidemic in the area.

## Data Availability

All data generated or analyzed during this study are included in this published article. Moreover, the datasets used and/or analyzed during the current study are available via an official request from Mashhad University of Medical Sciences.

## List of Abbreviations

HIV: Human Immunodeficiency Virus
HIV-RDT: HIV Rapid Diagnostic Test
MSM: Men Have Sex with Men
FSW: Female Sex Worker
MTCT: Mother-to-Child Transmission
MENA: Middle East and North Africa
TB: Tuberculosis
ACF: Active Case Finding
PHC: Public Health Care
VCT: Voluntary Counseling and Testing
